# Predicting dry weight change in Hemodialysis patients using machine learning

**DOI:** 10.1186/s12882-023-03248-5

**Published:** 2023-06-29

**Authors:** Hiroko Inoue, Megumi Oya, Masashi Aizawa, Kyogo Wagatsuma, Masatomo Kamimae, Yusuke Kashiwagi, Masayoshi Ishii, Hanae Wakabayashi, Takayuki Fujii, Satoshi Suzuki, Noriyuki Hattori, Narihito Tatsumoto, Eiryo Kawakami, Katsuhiko Asanuma

**Affiliations:** 1 Department of Nephrology, Graduate School of Medicine, Chiba University, Chuo-ku, Chiba, Japan; 2 Department of Artificial Intelligence Medicine, Graduate School of Medicine, Chiba University, Chuo- ku, Chiba, Japan; 3 Advanced Data Science Project (ADSP), RIKEN Information R&D and Strategy Headquarters, RIKEN, Yokohama, Kanagawa Japan; 4 Department of Artificial Kidney, Chiba University Hospital, Chuo-ku, Chiba, Japan; 5 Department of Nephrology, Seirei Sakura Citizen hospital, Sakura, Chiba Japan; 6 Department of Emergency and Critical Care Medicine, Graduate School of Medicine, Chiba University, Chiba, Japan

**Keywords:** Machine learning, Random Forest classifier, Importance analysis, Dry Weight, Hemodialysis

## Abstract

**Background:**

Machine Learning has been increasingly used in the medical field, including managing patients undergoing hemodialysis. The random forest classifier is a Machine Learning method that can generate high accuracy and interpretability in the data analysis of various diseases. We attempted to apply Machine Learning to adjust dry weight, the appropriate volume status of patients undergoing hemodialysis, which requires a complex decision-making process considering multiple indicators and the patient’s physical conditions.

**Methods:**

All medical data and 69,375 dialysis records of 314 Asian patients undergoing hemodialysis at a single dialysis center in Japan between July 2018 and April 2020 were collected from the electronic medical record system. Using the random forest classifier, we developed models to predict the probabilities of adjusting the dry weight at each dialysis session.

**Results:**

The areas under the receiver-operating-characteristic curves of the models for adjusting the dry weight upward and downward were 0.70 and 0.74, respectively. The average probability of upward adjustment of the dry weight had sharp a peak around the actual change over time, while the average probability of downward adjustment of the dry weight formed a gradual peak. Feature importance analysis revealed that median blood pressure decline was a strong predictor for adjusting the dry weight upward. In contrast, elevated serum levels of C-reactive protein and hypoalbuminemia were important indicators for adjusting the dry weight downward.

**Conclusions:**

The random forest classifier should provide a helpful guide to predict the optimal changes to the dry weight with relative accuracy and may be useful in clinical practice.

**Supplementary Information:**

The online version contains supplementary material available at 10.1186/s12882-023-03248-5.

## Background

Machine learning (ML) has been increasingly used in the medical field for diagnosing and predicting illnesses based on multiple parameters and background characteristics of patients [1]. ML has demonstrated a pivotal role in estimating the onset of acute kidney injury [2–5] and the therapeutic responses of diabetic nephropathy [6] and IgA nephropathy [7]. In dialysis treatment, ML has also shown to be useful in the adjustment of the erythropoiesis-stimulating agent dosage for renal anemia [8–11], prediction of the occurrence of hypotension [12–14], and evaluation of fluid volume for patients undergoing dialysis [15]. In healthcare systems, ML has the potential to improve the selection of appropriate investigation and therapeutic processes, possibly resulting in improved prognosis in hemodialysis patients.

Heart failure is a major cause of mortality among patients undergoing hemodialysis [16]. Many laboratory and clinical parameters are associated with the incidence and progression of heart failure, including hypertension, anemia, serum calcium levels, and excess extracellular volume [17]. Among these, dry weight (DW) is pivotal in controlling the volume status in hemodialysis patients [17]. DW is defined as “body weight with adequate fluid volume without excessive hypotension during dialysis and with minimal cardiovascular burden in the long term [18].” Therefore, maintaining an appropriate DW is crucial for the prevention of heart failure and results in the reduction of mortality in hemodialysis patients [19].

Clinically, physicians determine DW by considering multiple indicators, such as blood pressure, increased body weight between dialysis sessions, cardiothoracic ratio, pleural effusion, edema, brain natriuretic peptide (BNP), and other blood tests [20]. Revising and changing a patient’s DW according to their condition, including infectious diseases, diet, and physical activity at the time, is necessary. That is, DW cannot be determined by simple calculation, without specific indicators, and the weight of each parameter varies per patient. Trends in fluid volume changes may exist with changes in appetite, physical activity, and acute illness but are challenging to predict accurately. Therefore, automating information collection and processing, supporting diagnosis, and treatment using ML is urgently needed.

The random forest (RF) classifier is a ML method that generates high accuracy in the data analysis of various diseases, such as cardiovascular disease [21], stroke [22], cataracts [23], and ovarian cancer [24], because it can consider interactions between variables and is not affected by possible outliers. The RF classifier is one of the class identification methods in which data and explanatory variables are randomly divided to create multiple decision trees, and the final classification is achieved by the majority vote [25]. Because the correlations between each decision tree are weakened, overfitting is suppressed, which improves prediction performance. In addition, the RF classifier has high interpretability, such as the ability to calculate the relative importance of input variables, and is considered helpful for cases with many explanatory variables.

Previous studies using ML have shown that there are significant errors in the predictions of DW [26, 27]. Therefore, in the present study, we aimed to apply an RF classifier to predict the adjustments of DW made by dialysis specialists using many explanatory variables.

## Methods

### Patients and variables

**Fig. 1:**
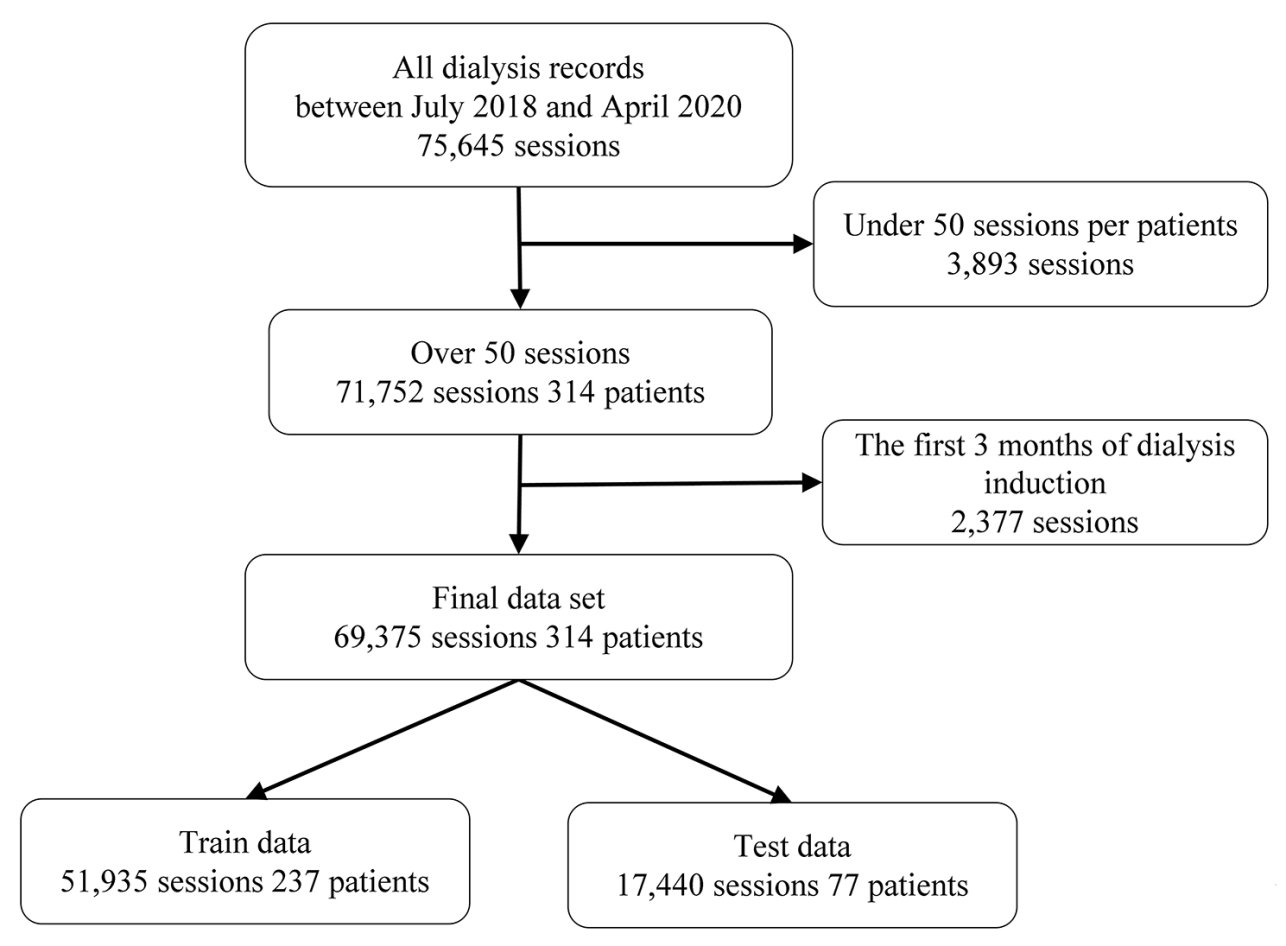
Process of selecting the subjects

We conducted a retrospective observational study at the Seirei Sakura Citizen Hospital in Chiba, Japan. Figure 1 shows the subject selection process. The study included patients who underwent hemodialysis at the facility twice or thrice a week for at least three months and were aged 20 years or older. All dialysis records were extracted from the dialysis system (Nikkiso Co., Ltd., Tokyo, Japan) from July 2018 to April 2020. A unique format was created to extract dialysis records from the dialysis system. The records were aggregated for each patient, and patients with more than 50 dialysis records were selected. The dialysis records within the first three months from the initiation of dialysis were excluded because of the unstable volume status in that period. CHDF and apheresis sessions are excluded to ensure consistency of conditions.

The clinical parameters, backgrounds, and medications of the eligible patients were collected from the electronic medical record system and anonymized. Laboratory tests were routinely performed twice a month at the beginning of the week. Table 1 lists the variables used in the extracted data.

**Table 1:** Input variables used in analyses

category	items
Demographic and anthropometric data	Age, gender, race, height, date of introduction of dialysis, name of primary disease
Laboratory test results	Pre- and post-dialysis WBC, RBC, Hb, Ht, Plt, Ret, TP, Alb, ALP, ChE, BUN, Cre, Na, K, Cl, Ca, IP, intact PTH, Fe, UIBC, FER, BNP, CRP
Prescription data	Drug name, dosage, number of days prescribed, and start date
Medical record entries that include the following keywords	Pulmonary congestion, pleural effusion, oxygen administration, edema, hypotension
Dialysis record	Membrane area of dialyzer, blood flow rate, injected drugs during dialysis, cardiothoracic ratio, median values of vital signs (blood pressure, pulse rate, body temperature), intradialytic hypotension

WBC, white blood cell; RBC, red blood cell; Hb, hemoglobin; Ht, hematocrit; Plt, platelet; Ret, reticulocyte; TP, total protein; Alb, albumin; ALP, alkaline phosphatase; ChE, cholinesterase; BUN, blood urea nitrogen; Cre, creatinine; Na, sodium; K, potassium; Cl, chlorine; Ca, calcium; IP, inorganic phosphorus; PTH, parathyroid hormone; Fe, iron; UIBC, unsaturated iron binding capacity; FER, ferritin; BNP, brain natriuretic peptide; CRP, C-reactive protein

### Dialysis and dry weight

Generally, patients undergo hemodialysis twice or thrice a week for 240 min per session. In every session, nephrologists are attending near the hemodialysis patients and checking the hemodialysis procedure being correctly done. Hemodialysis patients usually have regular laboratory tests twice a month, meaning they are tested every two or three weeks. The DW of each patient was assessed and adjusted by several nephrologists periodically and when clinically indicated, by referring to multiple indicators such as blood pressure, clinical findings, chest x-ray findings, laboratory test results, sonographic measurement of the inferior vena cava diameter, and blood volume monitor, as recommended by the Japanese Society for Dialysis Therapy guidelines [18] and K/DOQI clinical practice guidelines [28].

The default dialysate sodium concentration was 140 mmol/L, and the dialysate calcium concentration was 2.75 mmol/L. The dialysate flow rate was 500 ml/min, and the dialysate temperature was 36.0 °C for almost all patients.

### Data preprocessing

Table 1 shows the list of input variables used in this analysis. The pre-dialysis blood test results are used from the day of the test until the day before the next blood test. The post-dialysis laboratory test results are used as the data of the following dialysis date and are valid until the next test is performed. Each drug in the prescription data was divided into categories based on its efficacy, and the top 50 categories with the highest number of prescriptions were assigned an identification code. We manually confirmed the presence or absence of clinical findings from medical record entries containing the following keywords: pleural effusion, edema, oxygen demand, and hypotension. Dialyzers were expressed as membrane areas. Median values were obtained from all vital signs (including blood pressure and heart rate) measurements during each dialysis session and is only used on the same day. Intradialytic hypotension was defined as a fall in systolic blood pressure of 20 or more during a dialysis session. Among the above, dummy variables were used, except for continuous variables. The missing values were filled with the previous or average values before splitting the train and test set (Supplementary Table S1). 75% of the data (237 patients) were randomly selected for the training dataset, and the remaining 25% (77 patients) were used as the test dataset to evaluate the performance of the prediction. In the dataset, physicians adjusted the DW upward (downward) from the previous session in approximately 2.5% (3.0%) of all dialysis sessions. Whether these changes were made was used as the objective variable in machine learning.

### Supervised machine learning and importance analysis

There are many machine learning algorithms that can be applied to this task. In our initial examination, we have found that RF produces better results than other algorithms such as XGBoost and ANN, since the number of explanatory variables in our data is not very large and the task is a simple binary prediction of Dry Weight change. Our previous study also showed that RF performs reproducibly better than other algorithms in a binary classification task based on the similar clinical parameters as in this study [24]. For these reasons, we have applied RF classifier to train the models to predict the change in DW. The RF is an ensemble learning method that improves performance by training multiple models. In the RF models, training is performed using multiple decision trees. In the training process, the dataset was randomly selected for each decision tree using bootstrap sampling. The RF prediction is achieved by taking the majority vote of each decision tree. At each dialysis session, two RF classifier models were trained separately to determine whether “the DW should be adjusted upward or not” and “the DW should be adjusted downward or not,” because we hypothesized that the factors involved in Dry Weight increase were different from those involved in Dry Weight decrease, and it would be important to build separate models to account for the different contributions of the explanatory variables. The label data were set as the actual DW change by the nephrologists of the hospital. The RandomForestClassifier in the Python package scikit-learn was used in the analysis. GridSearchCV was applied to automatically optimize the hyperparameters of the RF classifier. For hyperparameter optimization, we use the following hyperparameters candidates for two models: max_depth: [None, 2, 3, 4, 5, 6], max_features: [“auto”, 6, 12, 24, 48], criterion: [“gini”, “entropy”]. The labels of the dataset in this study were imbalanced (the number of sessions “without changing DW” was relatively high compared to the number of sessions “adjusting DW upward” or “downward”). To regulate this imbalance, the Synthetic Minority Over-sampling Technique (SMOTE) algorithm was applied [29]. SMOTE is a method of oversampling that increases the minority population of imbalanced data. SMOTE in the Python package imbalanced learning was used in this study. For each dialysis session for each patient, the two models predicted the probabilities of “the DW should be adjusted upward” and “the DW should be adjusted downward,” denoted as “P_up_” and “P_down_” scores, respectively, with both scores ranging between 0 and 1. The probabilities of the classified predictions were rescaled using the probability calibration [30–32]. The trained RF classifier models were calibrated using CalibratedClassifierCV in the scikit-learn package. The overall performance of each model was evaluated using an area under the curve (AUC) of receiver operating characteristic (ROC) curve for the test data set. We also calculated accuracy, precision, recall, and F1 at the optimal probability threshold with the highest Youden Index. To monitor changes in a patient, we examined the probability changes for each patient. In addition, the predicted probability changes were evaluated in the 30 hemodialysis sessions before and after the DW change. The feature importance was obtained from the trained models, which show the contribution of each feature to the prediction.

## Results

### Patient characteristics

We retrospectively collected 69,375 dialysis records from 314 Asian patients during the observation period. The average observation period was 17.5 months. 73% of the patients were men. The mean age of the patients was 66.4 years old, and the median dialysis vintage was 4.0 years. The most common primary disease for dialysis induction was diabetic nephropathy (42.4%), followed by chronic glomerulonephritis (19.4%), nephrosclerosis (16.6%), and others (17.8%) (Table 2).

**Table 2:** Characteristics of patients and dialysis parameters in the training and test data

	All (n = 314)			Train data (n = 237)		Test data (n = 77)	
**Demographics**							
Age, years	66.4		± 12.4	66.4	± 12.5	66.3	± 12.3
Gender (Male/Female)	224	/90		170	/67	54	/23
Race, n (%)							
Asian (Japanese)	312	(99.4)		235	(99.2)	77	(100.0)
Asian (Southeast Asian)	2	(0.6)		2	(0.8)	0	(0.0)
Dialysis vintage, years	4.0	(2.0–8.0)		4.0	(2.0-8.8)	4.0	(2.0–6.0)
Body mass index, kg/m2	22.2		± 4.3	22.1	± 3.8	22.6	± 5.4
**Primary disease, n (%)**							
Diabetic nephropathy	133		(42.4)	95	(40.1)	38	(49.4)
Chronic glomerulonephritis	61		(19.4)	51	(21.5)	10	(13.0)
Nephrosclerosis	52		(16.6)	40	(16.9)	12	(15.6)
Polycystic kidney disease	12		(3.8)	11	(4.6)	1	(1.3)
Others	56		(17.8)	40	(16.9)	16	(20.8)
**Dialysis parameter**							
UF (L/session)	2.4		± 0.97	2.4	± 0.95	2.6	± 1.03
UFR (L/hr)	0.61		± 0.24	0.60	± 0.24	0.64	± 0.25
Dry Weight (kg)	59.7		± 14.2	59.3	± 12.8	60.8	± 17.6
**Dialysis mode, n (%)**							
HD	5047		(7.3)	3238	(6.2)	1809	(10.4)
HD + ECUM	306		(0.4)	146	(0.3)	160	(0.9)
OHDF	63,700		(91.8)	48,320	(93.0)	15,380	(88.2)
OHDF + ECUM	254		(0.4)	185	(0.4)	69	(0.4)
ECUM	68		(0.1)	46	(0.1)	22	(0.1)

Values are expressed as mean ± standard deviation, median (interquartile range), or percent frequency

UF, ultrafiltration; UFR, ultrafiltration rate; HD, hemodialysis; ECUM, extracorporeal ultrafiltration method; OHDF, online hemodiafiltration

### Prediction of dry weight change with RF classifiers

To learn the physician’s decision on how to set the DW for the next dialysis session, two RF classifier models were trained separately with 51,935 dialysis records from 237 patients and validated for accuracy using 17,440 records from 77 patients.

**Fig. 2:**
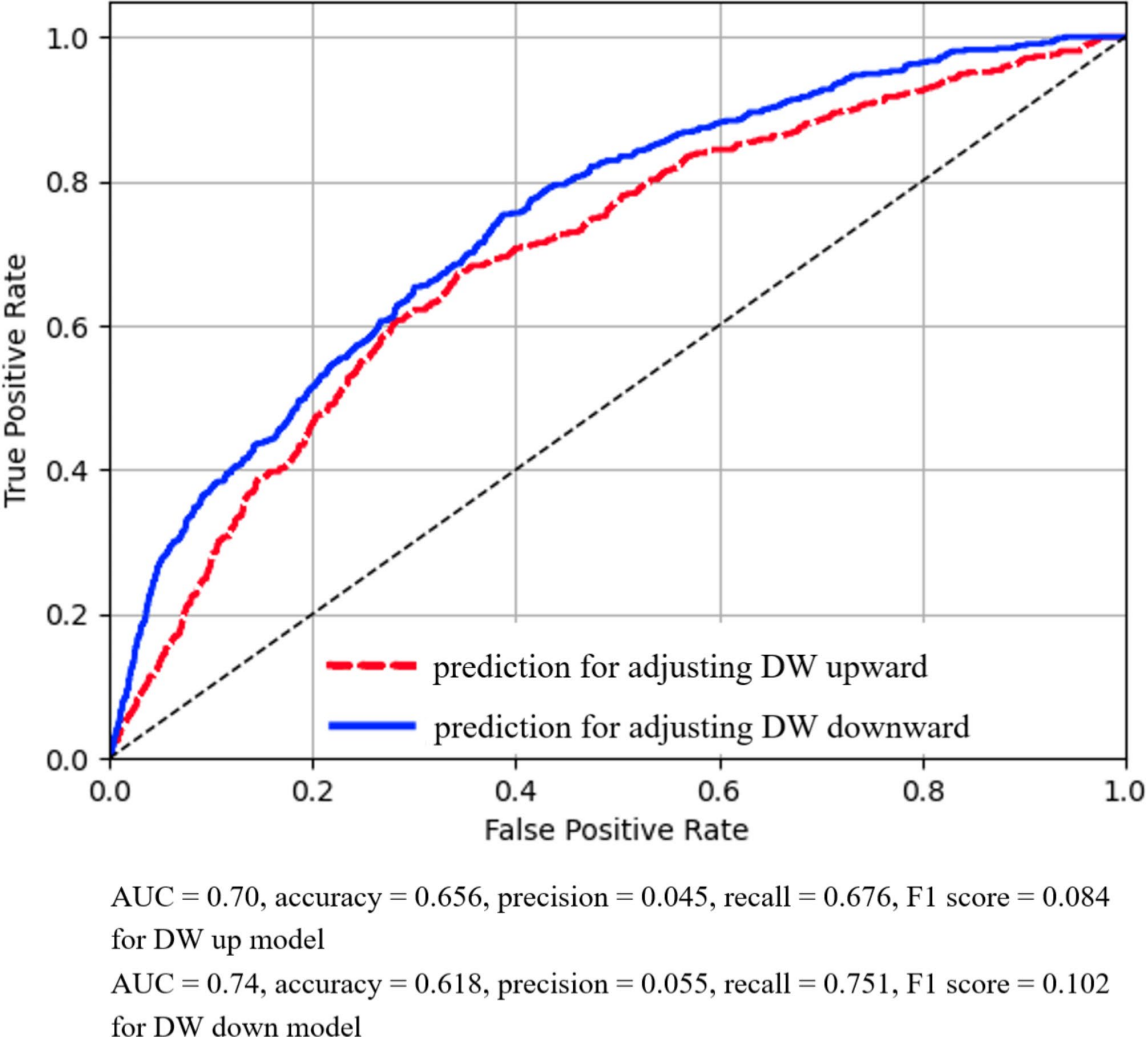
ROC curves of the ML models for the test data set The dashed line and solid line indicate the ROC curves of the models for the prediction of an upward and downward change in DW, respectively ROC, receiver operating characteristic; ACU, area under the curve; DW, dry weight

**Fig. 3:**
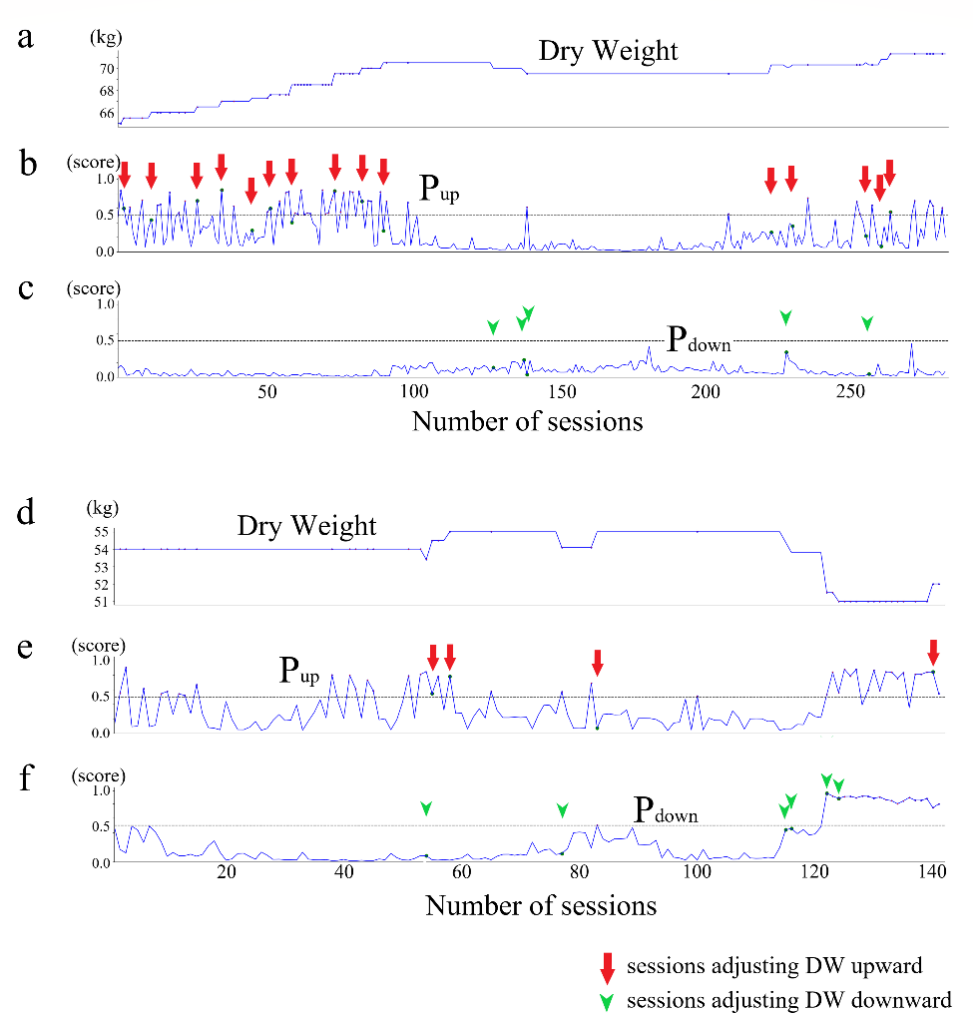
Representative examples of prediction **(a–c)** and **(d–f)** shows the representative examples of two patients from the test data set **(a)** and **(d)** show the actual DW trend; **(b)** and **(e)** show the P_up_ scores; **(c)** and **(f)** show the P_down_ scores Arrows and arrowheads indicate sessions where the DW was adjusted upward and downward Horizontal axis: the number of dialysis sessions along the time Vertical axis: weight in kilograms in **(a)** and **(d)**, the probability value from 0 to 1 in **(b)**, **(c)**, **(e)**, and **(f)**

As the result of GridsearchCV, we got (max_depth = 3, max_features = 12, criterion = “gini”) for DW up model, and (max_depth = 5, max_features = 12, criterion = “entropy”) for DW down model. After the calibration of the models, the optimal probability thresholds with the highest Youden were 0.204 for the DW up model, and 0.114 for the DW down model. The AUC, accuracy, precision, recall, and F1 score of the two models for the test data set were (AUC = 0.70, accuracy = 0.656, precision = 0.045, recall = 0.676, F1 score = 0.084) for DW up model, and (AUC = 0.74, accuracy = 0.618, precision = 0.055, recall = 0.751, F1 score = 0.102) for DW down model, respectively (Fig. 2). We also examined the relationship between the actual DW changes and the predicted probabilities of the ML models, P_up_ and P_down_, in two representative cases. In the first case, shown in Fig. 3a and b, and 3c, the machine learning prediction, P_up_, was greater than 0.5 in the majority of instances when the DW actually changed upward. P_down_ was often higher before and after the DW was changed downward, but the values were smaller than those of P_up_ and often did not show a clear peak. In the second case, shown in Fig. 3d and e, and 3f, the actual DW remained fixed at a constant value despite the many P_up_ peaks in the first half of the case. The P_down_ was low in the first half, but persistently quite high, close to 1.0, around the end of the recording. Interestingly, P_up_ was also quite high during this period, indicating a conflict between the two machine learning models.

**Fig. 4:**
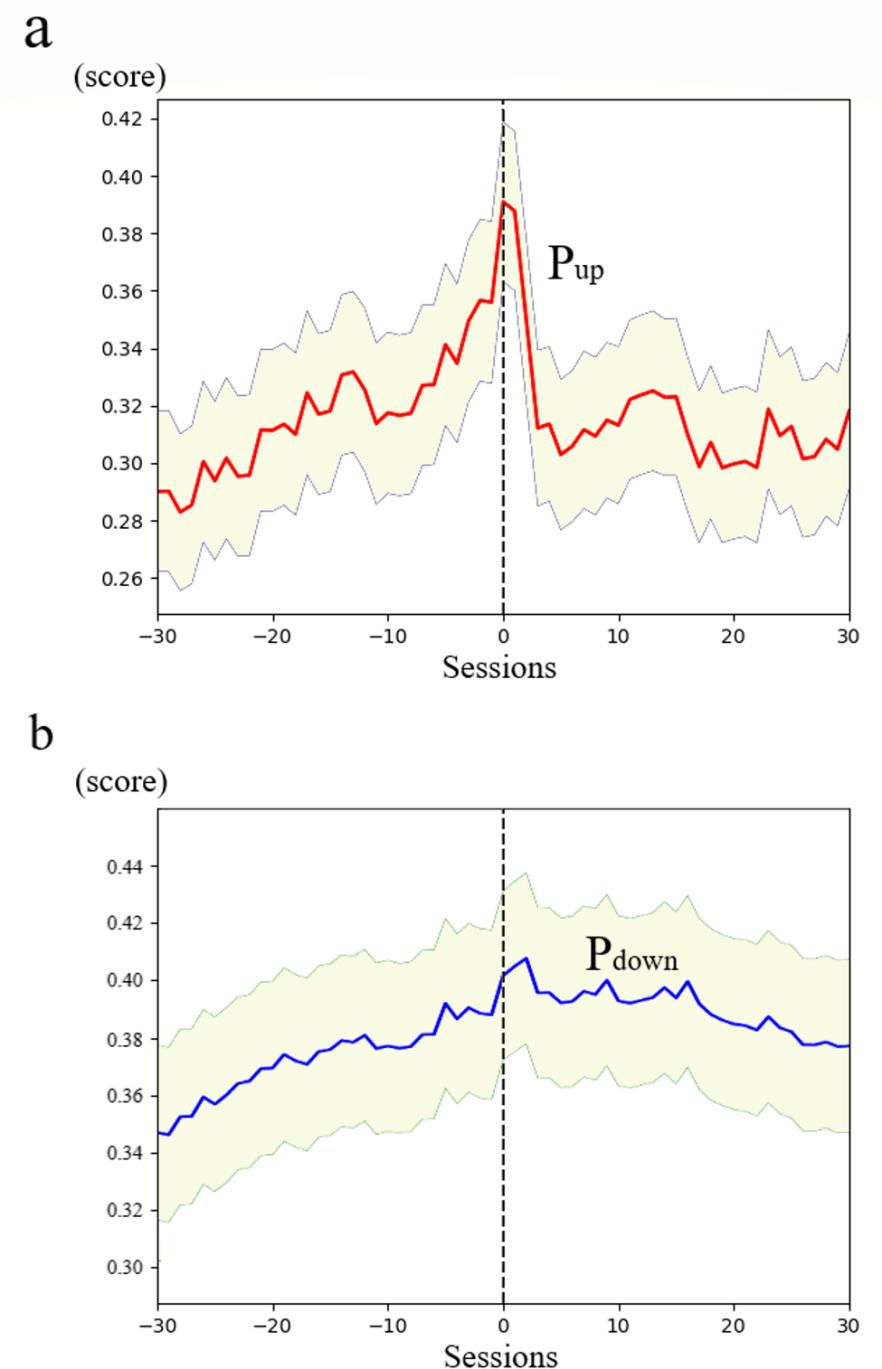
The behavior of P_up_ and P_down_ scores around the actual DW changes in the test dataset **(a)** Average P_up_ score and **(b)** average P_down_ score 30 sessions before and after the actual DW changes, represented as session 0. Even if a patient’s DW was changed in successive sessions, each change was treated separately. Shaded areas indicate 95% confidence intervals Horizontal axis: the number of dialysis sessions along the time Vertical axis: the probability value from 0 to 1

Thereafter, we examined the changes in the prediction probabilities of ML in the 30 hemodialysis sessions before and after the actual DW change for all cases included in the test dataset. The average P_up_ had a sharp peak at the time of the actual upward change in DW and then declined immediately (Fig. 4a). However, the change in P_down_ was slow and remained high for a while after the actual downward change in DW occurred (Fig. 4b).

### Identification of key factors in the prediction of DW changes

**Fig. 5:**
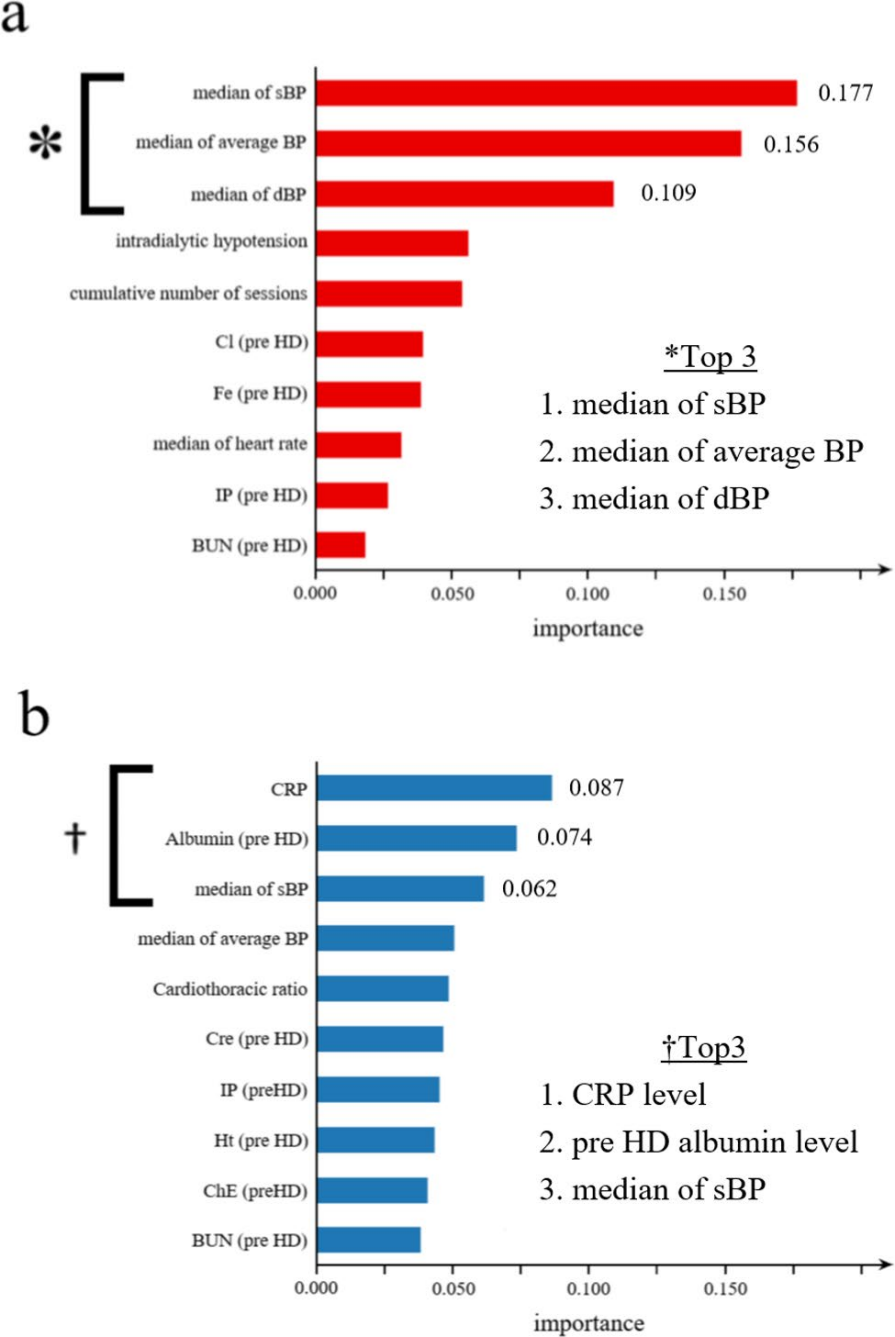
Variable importance of the two ML models The relative importance of input variables **(a)** in the model to predict an upward DW change and **(b)** in the model to predict a downward DW change Horizontal axis: relative importance Vertical axis: input variables listed in order of importance values sBP, systolic blood pressure; dBP, diastolic blood pressure; CRP, C-reactive protein; HD, hemodialysis

To clarify the parameters on which the determination of DW changes in the ML process is based, we calculated the variable importance of the two ML models. Figure 5 shows the relative importance of the input variables in each model, listed in order of importance values.

For the model to predict upward DW changes, variables related to blood pressure are of great importance (Fig. 5a). The top three most important variables were the median value of systolic blood pressure, mean blood pressure, and diastolic blood pressure measured several times during a single dialysis session.

Systolic blood pressure was also an essential factor in predicting downward DW changes (Fig. 5b). However, the two most important variables were C-reactive protein (CRP) and pre-dialysis albumin levels, while blood pressure was relatively less important.

**Fig. 6:**
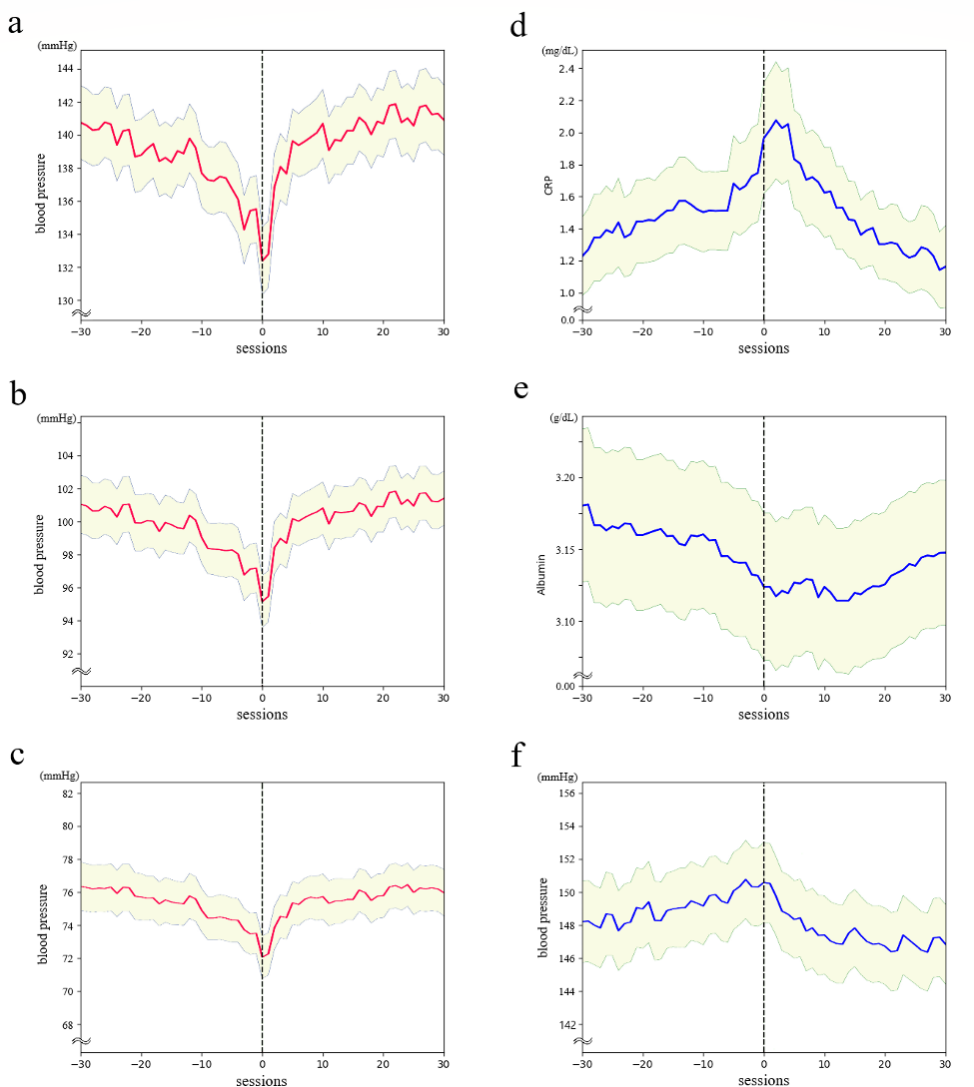
The trend in the most important input variables around the actual DW changes The trends of the top three most important input variables in the model to predict an upward DW change (a–c) and predict a downward DW change (d–f) **(a)** median systolic blood pressure, **(b)** median average blood pressure, and **(c)** median diastolic blood pressure declined at the time of the upward DW change **(d)** CRP level was elevated, **(e)** pre-dialysis albumin level declined, **(f)** median systolic blood pressure was slightly elevated at the time of the downward DW change. Shaded areas indicate 95% confidence intervals Horizontal axis: the number of dialysis sessions along the time sBP, systolic blood pressure; dBP, diastolic blood pressure; CRP, C-reactive protein; HD, hemodialysis

Next, the transition of the most important features was examined in 30 hemodialysis sessions before and after the actual DW change. Blood pressure, an important predictor of the upward DW change, showed a marked decrease at the time of the actual upward DW change, followed by rapid recovery (Fig. 6a and b, and 6c). However, even the systolic blood pressure, which declined the most, only dropped from 141 mm Hg to 133 mm Hg on average over 30 dialysis sessions.

CRP, an important indicator of the downward DW change, showed a sharp increase just before the actual DW change, but its peak occurred a little later than the actual DW change (Fig. 6d). The change in albumin was slower and began to increase immediately after the actual DW change (Fig. 6e). Systolic blood pressure, which showed a sharp peak around the upward DW change, showed a slow change before and after the downward DW change (Fig. 6f). CRP levels rose from 1.2 mg/dL to 2.1 mg/dL, albumin levels declined from 3.18 g/dL to 3.12 g/dL, and the median systolic blood pressure rose slightly from 148 mm Hg to 150 mm Hg on average over 30 dialysis sessions.

## Discussion

In this study, we developed novel methods to predict whether DW should be adjusted at each dialysis session using an RF classifier. The AUCs of the models were 0.70 or more, indicating high reliability. By analyzing approximately 150 variables, our approach revealed the most important input factors for each decision-making. These models may enable medical staff to determine the correct timing for adjusting DW more efficiently.

Several studies have used machine learning to predict DW in patients undergoing dialysis. Guo et al. predicted DW using a neural network model and compared it to the DW predicted by a body composition monitor; their model’s root mean square error was 1.316 [26]. Kim et al. used XGBoost machine learning to predict DW and compared it with DW based on bioimpedance spectroscopy [27]. If the difference between the two groups was between 1 and 2 kg, the average accuracies were 72–83%. Both results suggest the usefulness of machine learning. However, challenges remain, with significant errors in the prediction of the DW itself. Therefore, we focused on adjusting the DW upward or downward, rather than estimating the value of DW.

DW could be acceptable in a particular range for stable maintenance hemodialysis patients with few cardiac problems. Usually, DW is not immediately determined as a constant value but is probed and adjusted gradually based on various information gathered [20]. Thus, we believe our model helpful in informing us when the current dry weight may be out of the appropriate range and our approach has the advantage of immediate clinical application.

We have also shown how the predicted scores varied over time, another point that has yet to be addressed. Our models can capture even small changes in the input variables that reflect the patient’s condition.

The average P_up_ score showed a quick rise, precisely detecting the timing at which the DW should be adjusted upward. However, the average P_down_ score gradually changed. A possible reason is that the model could not catch up with real-time changes in medical conditions because the frequency of laboratory tests was only twice a month. Furthermore, a gradual reduction of DW is recommended in clinical settings [33], corresponding to the gentle, slow slope of the average P_down_ score.

Importance analysis revealed that serum levels of CRP and albumin, rather than blood pressure, showed the highest importance in predicting DW reduction. In addition to the elevation of CRP, hypoalbuminemia also indicates an inflammatory status [34, 35], which is related to a decrease in muscle mass [36] and an increase in extracellular volume [37, 38] in patients. Many studies have reported a strong association between inflammation and overhydration [39–41], which can lead to heart failure. Additional studies have shown that inflammation is associated with mortality [42–44]. Although these are notable findings, using ML clearly indicates that an inflammatory status is the most important in adjusting DW downward.

Importance analysis also showed that declining blood pressure was related to predicting upward changes in DW. Several studies have shown that hypotension during dialysis is a significant poor prognostic factor [45, 46]. Decreased blood pressure can lead to a higher risk of falls, stroke, arteriovenous shunt occlusion, and so on [47–49]. Therefore, to prevent hypotension during dialysis, the ability to notify medical staff of the appropriate time that the DW should be adjusted upward is useful.

In our data, there were only small changes in the average levels of CRP, albumin, and blood pressure in the sessions where DW was actually adjusted, compared to 30 previous sessions. Even if these changes are gradual and difficult for medical staff to recognize, our ML models can detect slight changes and inform them by raising their scores. Using our models, DW can be adjusted to prevent adverse events and improve a patient’s quality of life and prognosis.

BNP has been used to indicate fluid volume status, cardiac function, and cardiovascular disease. Many reports have shown an association between the biomarker and heart failure and prognosis in hemodialysis patients, but the threshold for this association is not consistent [50]. In the current analysis, the importance of BNP was not ranked high. This was most likely because BNP is measured too infrequently to be used to predict DW adjustment at every dialysis session. Furthermore, cardiac function and cardiovascular disease can significantly change the BNP value even in the same fluid volume status, so BNP may not be effectively used in our model.

For each patient, the predicted scores were elevated around the time of the actual DW changes in most cases; however, in some instances, both the P_up_ and P_down_ scores were elevated simultaneously. Most of them were as follows: the elevation of a P_down_ score caused the reduction of DW and was immediately followed by a drop in blood pressure and hence an increasing P_up_ score, as well as elevated inflammatory states and low blood pressure in critical conditions. Our models can detect these unstable situations.

In a real clinical setting, based on inputs such as vital signs, clinical findings and blood test results of each patient, our model could calculate the probabilities that DW should be raised and lowered respectively. If the probability exceeds thresholds, alerts will be issued, prompting the doctor to consider changing the DW. The alerts are also helpful for non-specialist doctors, other medical staff, and trainees. Furthermore, our model can be presented to patients as a rationale for changing their DW.

Our study has several limitations. Due to the retrospective nature of this study, the description of some findings, such as edema and pleural effusion, seemed inadequate in some cases. In addition, because of the low frequency of laboratory tests, the models do not reflect real-time changes. Therefore, we plan to start collecting data prospectively, using templates to enter findings and devices to monitor blood concentration during dialysis to improve the accuracy of our models. Furthermore, the findings of this work are not immediately generalizable because it was a single-center study. In the future, we plan to collect data from other dialysis facilities and apply transfer learning to them.

## Conclusions

In summary, we developed novel models to predict whether the DW of hemodialysis patients should be adjusted using an RF classifier. Our analysis showed the importance of declining blood pressure during dialysis for predicting an upward change in the DW and the impact of elevated CRP levels and hypoalbuminemia on predicting a downward change in the DW. Further studies are required to evaluate the clinical effectiveness of our models.

## Electronic supplementary material

Below is the link to the electronic supplementary material.

Supplementary Material 1

## Data Availability

The data underlying this article cannot be shared publicly due to the privacy of individuals that participated in the study. The data will be shared upon reasonable request to the corresponding author.

## Abbreviations

ML: Machine learning
DW: Dry weight
BNP: Brain natriuretic peptide
RF: Random forest
SMOTE: Synthetic Minority Over-sampling Technique
ROC: Receiver operating characteristic
AUCs: Area under the receiver operating curves
CRP: C-reactive protein
