# Supplementary material for: Predicting dry weight change in Hemodialysis patients using machine learning
Source: BMC Nephrol. 2023 Jun 29;24:196. doi: 10.1186/s12882-023-03248-5 (PMC10308746; doi:10.1186/s12882-023-03248-5)
Supplement: Supplementary file 1 — Supplementary Material 1 [file 12882_2023_3248_MOESM1_ESM.pdf]

Table S1. Missing values for each variable.

	All (n=314)		Train data (n=237)		Test data (n=77)	
**Dialysis records**						
Body weight before dialysis	0.0064		0.0063		0.0060	
Body weight after dialysis	0.0064		0.0063		0.0059	
Water removal	0.0067		0.0067		0.0063	
Membrane area of dializer	0.0001		0.0001		0.0000	
Dialysis time	0.0064		0.0063		0.0059	
Blood flow rate	0.0065		0.0064		0.0061	
Cardiothoracic ratio	0.0435		0.0433		0.0435	
Blood pressure	0.0065		0.0065		0.0059	
Pulse rate	0.0065		0.0065		0.0059	
Body temperature	0.8058		0.8029		0.8145	
**Medical record entries**						
Pulmonary congestion	0.9995		0.9994		0.9998	
Pleural effusion	0.9985		0.9987		0.9979	
Oxygen administration	0.9995		0.9995		0.9995	
Edema	0.9954		0.9957		0.9944	
Hypotension	0.9999		0.9999		0.9999	
**Laboratory test results**	(pre-HD)	(post-HD)	(pre-HD)	(post-HD)	(pre-HD)	(post-HD)
White blood cell count	0.8258	0.9999	0.8270	0.9999	0.8221	0.9999
Red blood cell count	0.8258	0.9999	0.8270	0.9999	0.8221	0.9999
Hemoglobin	0.9291	0.9999	0.9292	0.9999	0.9288	1.0000
Hematocrit	0.8258	0.9999	0.8270	0.9999	0.8221	0.9999
Platelet count	0.8258	0.9999	0.8270	0.9999	0.8221	0.9999
Reticulocytes	0.9251	N.A.	0.9251	N.A.	0.9249	N.A.
Total protein	0.8390	0.9999	0.8395	0.9999	0.8372	1.0000
Albumin	0.8378	0.9999	0.8387	0.9999	0.8349	1.0000
Alkaline phosphatase	0.9143	1.0000	0.9146	0.9999	0.9135	1.0000
Cholinesterase	0.9511	N.A.	0.9519	N.A.	0.9485	N.A.
Blood urea nitrogen	0.8381	0.9272	0.8384	0.9272	0.8372	0.9273
Creatinine	0.8381	0.9272	0.8384	0.9272	0.8372	0.9273
Sodium	0.8361	0.9272	0.8364	0.9272	0.8351	0.9272
Potassium	0.8361	0.9272	0.8364	0.9272	0.8351	0.9272
Chlorine	0.8361	0.9272	0.8364	0.9272	0.8351	0.9272
Calcium	0.8399	0.9272	0.8407	0.9272	0.8376	0.9273
Inorganic phosphorus	0.8416	0.9272	0.8427	0.9272	0.8381	0.9273
Intact parathyroid hormone	0.9262	N.A.	0.9263	N.A.	0.9261	N.A.
Iron	0.9257	N.A.	0.9257	N.A.	0.9257	N.A.
Unsaturated iron binding capacity	0.9260	N.A.	0.9261	N.A.	0.9259	N.A.
Ferritin	0.9259	N.A.	0.9260	N.A.	0.9257	N.A.
Brain natriuretic peptide	0.9960	0.9997	0.9957	0.9997	0.9968	0.9997
C-reactive protein (pre- or post-HD)	0.8916		0.8933		0.8862	

There are no missing value for age, gender, height, name of primary disease, prescription, injected drugs during dialysis, dry weight or dialysis mode. The dates of introduction of dialysis for three patients included in the train data were not available.

Values are expressed as ratio to the number of dialysis records. HD, hemodialysis.
